# Exploring Prognostic Factors and Survival Outcomes in Advanced Non-Small Cell Lung Cancer Patients Undergoing First-Line Chemotherapy in Limited-Resource Settings

**DOI:** 10.3390/jcm14020335

**Published:** 2025-01-08

**Authors:** Chawalit Chayangsu, Jiraporn Khorana, Chaiyut Charoentum, Virote Sriuranpong, Jayanton Patumanond, Apichat Tantraworasin

**Affiliations:** 1 Department of Internal Medicine, Surin Hospital, Institute of Medicine, Suranaree University of Technology, Surin 32000, Thailand; chawalit.sur@cpird.in.th; 2 Department of Surgery, Division of Pediatric Surgery, Faculty of Medicine, Chiang Mai University, Chiang Mai 50200, Thailand; nanji22@gmail.com; 3 Clinical Epidemiology and Clinical Statistic Center, Faculty of Medicine, Chiang Mai University, Chiang Mai 50200, Thailand; jpatumanond@gmail.com; 4 Department of Internal Medicine, Faculty of Medicine, Chiang Mai University, Chiang Mai 50200, Thailand; ccharoentum@gmail.com; 5 Department of Internal Medicine, Faculty of Medicine, Chulalongkorn University, Bangkok 10330, Thailand; vsmdcu40@gmail.com; 6 Clinical Surgical Research Center, Chiang Mai University, Chiang Mai 50200, Thailand

**Keywords:** non-small cell lung cancer, chemotherapy, prognostic factors, survival, restricted mean survival time, limited-resource

## Abstract

**Background/Objectives**: Non-small cell lung cancer (NSCLC) remains a leading cause of cancer-related mortality globally, especially in limited-resource countries (LRCs) where access to advanced treatments such as targeted therapy and immunotherapy is constrained. Platinum-based chemotherapy remains a cornerstone of first-line therapy. This study aims to identify prognostic factors influencing survival outcomes and evaluate treatment response to chemotherapy in advanced NSCLC patients in LRCs. **Methods**: A retrospective cohort study was conducted on 200 advanced NSCLC patients treated with first-line platinum-based doublet chemotherapy at Surin Hospital Cancer Center, Thailand. Prognostic factors were assessed through univariate and multivariate Cox regression analyses. Additionally, restricted mean survival time (RMST) was calculated to compare survival outcomes between responders and non-responders. **Results**: Independent prognostic factors associated with improved survival included good performance status, ECOG 0–1 (HR 0.50, *p* = 0.012), serum albumin ≥ 3.5 mg/dL (HR 0.60, *p* = 0.010), and favorable response to chemotherapy (HR 0.57, *p* = 0.003). Responders demonstrated significantly longer RMST at 12 months (*p* < 0.001), 24 months (*p* < 0.001), and 36 months (*p* = 0.004) compared to non-responders. **Conclusions**: Identifying prognostic factors and treatment responses is important for improving outcomes in advanced NSCLC patients, particularly in limited-resource settings where access to novel therapies is restricted.

## 1. Introduction

Non-small cell lung cancer (NSCLC) is the most common type of lung cancer and one of the leading causes of cancer-related mortality globally, accounting for approximately 85% of all lung cancer cases [1]. The disease is often diagnosed at an advanced stage, particularly in limited-resource countries (LRCs), where healthcare access barriers and limited diagnostic resources delay detection and intervention. As a result, advanced NSCLC frequently has a poor prognosis, with a median survival time of less than one year, even with treatment [2]. First-line treatment options for advanced NSCLC typically consist of platinum-based chemotherapy, either alone or in combination with immunotherapies or targeted therapies where resources allow [3].

In LRCs, however, the availability and affordability of advanced treatments vary significantly, posing challenges to implementing molecular testing and novel therapies like tyrosine kinase inhibitors (TKIs) and immune checkpoint inhibitors [4,5,6]. In settings such as Thailand, where health insurance schemes do not cover immunotherapies and only the first generation of EGFR TKIs has been accessible since 2021, traditional platinum-based chemotherapy remains the mainstay of treatment [7]. Platinum-based chemotherapy consists of a platinum agent, such as cisplatin or carboplatin, combined with another agent like paclitaxel, gemcitabine, or pemetrexed. As a first-line treatment, it typically achieves response rates of 20–30%, with a median progression-free survival of approximately 4–6 months and overall survival ranging from 8 to 12 months. This underscores the importance of optimizing available treatments and understanding prognostic factors that can better inform treatment decisions.

NSCLC is a highly heterogeneous disease, with survival influenced by factors such as patient performance status, nutritional health, cancer histology, and systemic inflammatory responses. Identifying key prognostic factors is essential to improve survival predictions and support treatment stratification, especially where access to novel therapies is limited [8,9]. Previous studies have highlighted the importance of factors such as Eastern Cooperative Oncology Group (ECOG) performance status, serum albumin levels, and body mass index (BMI); however, data from LRCs remain sparse. Given these circumstances, this study aims to evaluate prognostic factors and treatment responses in a cohort of advanced NSCLC patients receiving first-line chemotherapy in a limited-resource setting, comparing survival outcomes between responders and non-responders through restricted mean survival time (RMST).

## 2. Materials and Methods

A retrospective cohort study was conducted, including 200 patients with confirmed advanced NSCLC (stage IIIB or IV according to TNM 7th edition or recurrent disease with distant metastasis) treated with first-line platinum-based doublet chemotherapy from July 2014 to December 2018. Eligible patients were those aged 18 years or older, diagnosed with NSCLC confirmed by cytopathology, and treated with at least two cycles of platinum-based doublet chemotherapy (platinum plus paclitaxel or platinum plus gemcitabine). Only patients who completed a minimum of 2 cycles (4–6 cycles in total) were included to adequately assess treatment effects using computerized tomography (CT) scans conducted within one month. Patients with poor performance status (ECOG ≥ 3) and those scheduled for adjunctive radiotherapy were excluded. All included patients were followed through 31 January 2020, or until death, whichever came first, to obtain survival data.

Data were collected from medical records, including gender, age, smoking status, Eastern Cooperative Oncology Group (ECOG) performance status, history of weight loss greater than 5% in 3 months, body mass index (BMI), lung cancer histology, tumor grading based on official pathological reports, chemotherapy regimen, baseline laboratory data (serum albumin, hemoglobin, white blood cell (WBC) counts, and carcinoembryonic antigen (CEA) levels), and treatment response according to the Response Evaluation Criteria in Solid Tumors (RECIST) version 1.1 [10]. Patients with a complete response (CR) or partial response (PR) were classified as responders, and patients with stable disease (SD) or progressive disease (PD) were classified as non-responders. The primary outcome was to explore prognostic factors for survival, and the secondary outcome was to examine restricted mean survival time (RMST) between responders and non-responders. Survival time was measured from the date of the first day of chemotherapy to the date of death from any cause. The study protocol was reviewed and approved by the Ethics Committee of Surin Hospital, Ministry of Public Health of Thailand under protocol 23/2562 with an exemption from patient informed consent due to it being a full retrospective study. The study was registered by Thai Clinical Trials Registry number TCTR20220131001.

Statistical analysis was performed using the STATA version 16.0 Statistical Package. Data collection, design, and analysis in retrospective cohort studies are presented as mean (standard deviation), median (interquartile range), count, and percentage, as appropriate. Inferential statistics were conducted using the *T*-test and Kruskal–Wallis test for continuous variables, and Fisher’s exact test for categorical variables. Median survival time was calculated using the Kaplan–Meier method. Factors associated with survival time were explored using Cox regression analysis, reported as hazard ratio (HR) with a 95% confidence interval (CI). In addition to Cox proportional hazards modeling, RMST was calculated to provide an alternative survival measure, particularly valuable in settings where the proportional hazards assumption may not hold or where event rates are low. We compared RMST between responders and non-responders at pre-specified time points of 12, 24, and 36 months. The significance level was set at less than 0.05.

## 3. Results

A total of 200 advanced NSCLC patients were included in the study, with a slight majority being female (50.5%). The mean age was 62.4 years, and the majority (91.5%) presented with stage IV disease at the time of diagnosis. A smoking history was documented in 35.9% of patients, while 42.0% of the cohort was classified as underweight based on BMI criteria (<18.5 kg/m^2^). The most prevalent histology was adenocarcinoma (67.0%), followed by squamous cell carcinoma (26.3%). Additional details are summarized in Table 1.

The most common histology was adenocarcinoma (130, 67.0%), while squamous cell carcinoma and large cell carcinoma accounted for 26.3% and 0.5%, respectively. Additional details are summarized in Table 1.

### 3.1. Response and Outcome

**Figure 1:**
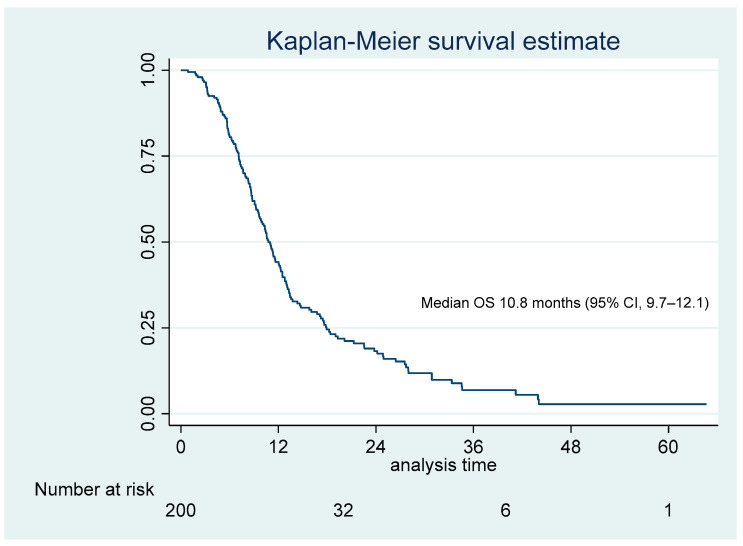
Kaplan–Meier curve for overall survival (OS) in 200 advanced NSCLC patients.

The platinum plus paclitaxel regimen was used more frequently (109 patients, 54.5%). Treatment response varied across patients, with an overall response rate (ORR) of 57.0%. Patients were categorized based on their treatment response per RECIST criteria: 2.0% achieved complete response (CR), 55.0% partial response (PR), 30.0% stable disease (SD), and 13.0% progressive disease (PD), as shown in Table 2. The median overall survival time for the entire cohort was 10.8 months (95% CI: 9.7–12.1), as shown in Figure 1.

### 3.2. Prognostic Factors

Univariate analysis of prognostic factors was performed for survival time. Good prognostic factors included performance status ECOG 0–1 (HR 0.43, *p* < 0.001), serum albumin ≥ 3.5 mg/dL (HR 0.55, *p* < 0.001), platinum plus gemcitabine chemotherapy regimen (HR 0.71, *p* = 0.027), and responder status (HR 0.60, *p* = 0.001). Cox regression analysis showed that ECOG 0–1 (HR 0.50, *p* = 0.012), serum albumin ≥ 3.5 mg/dL (HR 0.60, *p* = 0.010), and responder status (HR 0.57, *p* = 0.003) were independent prognostic factors for survival (Table 3).

### 3.3. Responders and RMST

**Figure 2:**
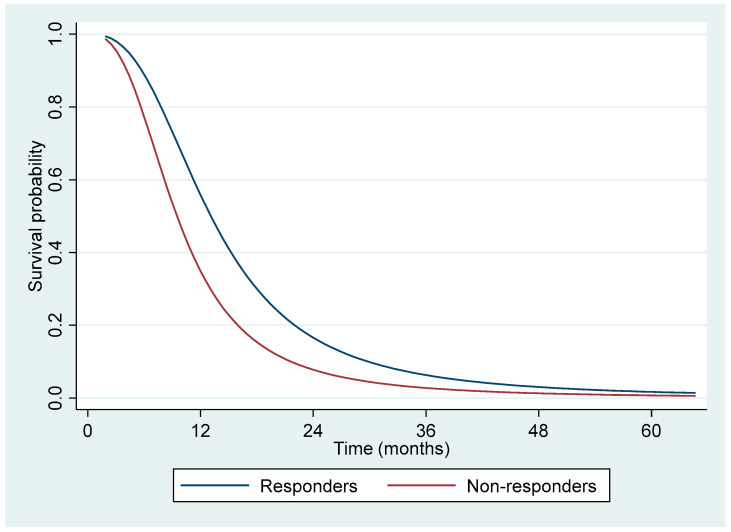
Restricted mean survival time over the follow-up period as the area under the survival curve.

To gain a more detailed understanding of survival beyond proportional hazards, we utilized RMST analysis, which provides an alternative perspective in survival studies. Patients who responded to chemotherapy demonstrated significantly longer RMST across three pre-specified time points: 12 months (+1.8 months, *p* < 0.001), 24 months (+3.6 months, *p* < 0.001), and 36 months (+4.2 months, *p* = 0.004). These findings underscore the substantial survival benefits associated with treatment response (Table 4 and Figure 2).

## 4. Discussion

The prognosis for patients with advanced non-small cell lung cancer (NSCLC) remains poor, particularly in settings where targeted therapy and immunotherapies are not accessible due to limited healthcare resources [11,12]. For such patients, conventional chemotherapy remains the primary treatment option due to a lack of molecular testing capabilities and the inability to afford expensive genetic testing or medications [4,5,7,11,13]. This emphasizes the need to understand clinical and pathological prognostic factors to optimize treatment decisions and allocate resources effectively.

In this retrospective study involving 200 advanced NSCLC patients treated with platinum-based doublet chemotherapy as first-line treatment, the overall response rate (ORR) was 57.0%, with a median survival time of 10.8 months. These findings align with those from other studies, such as Scagliotti et al. (2008), which reported a similar median survival of 10.3 months in patients treated with platinum-based regimens [14]. This consistency reinforces the role of platinum-based chemotherapy as an effective standard option in resource-limited settings.

Univariate and multivariate analyses identified several key prognostic factors for survival in advanced NSCLC. Performance status (PS) is widely recognized as a critical determinant of survival in NSCLC, and this study reaffirmed that a good PS (ECOG 0–1) significantly influences survival. This is consistent with existing literature [15], where PS not only correlates with response rates but also with overall survival, emphasizing its importance as a baseline measure for treatment decision making. Furthermore, serum albumin levels, an indicator of nutritional and inflammatory status, emerged as a significant prognostic factor [16]. Hypoalbuminemia has long been established as an adverse prognostic indicator in NSCLC [17,18], both independently and as part of composite prognostic scores such as the modified Glasgow Prognostic Score (mGPS) [9]. In our study, a pre-treatment serum albumin level ≥ 3.5 mg/dL was associated with favorable survival outcomes. Interestingly, our multivariate analysis did not identify some traditionally favorable factors, such as younger age, female gender [19], and higher body mass index (BMI) [20], as significant predictors of survival. While age and gender have been associated with differential survival outcomes in NSCLC, their impact may be less pronounced in advanced stages of the disease. This lack of association could indicate that, in later stages of NSCLC, performance status and other physiological factors, such as serum albumin, play a more critical role than demographic factors. Furthermore, while high BMI is often linked to improved survival in early-stage NSCLC, this advantage may diminish in advanced stages, where the disease exerts greater physiological strain.

The restricted mean survival time (RMST) analysis provided additional insights into treatment response, offering a reliable survival estimate beyond the limitations of traditional median survival calculations. RMST is particularly valuable in studies where proportional hazard assumptions do not hold or event rates are low. In this study, responders to chemotherapy exhibited significantly longer RMST at 12, 24, and 36 months compared to non-responders, demonstrating the sustained benefit of chemotherapy response in life expectancy. The robustness of RMST as an alternative survival measure can thus offer valuable guidance in clinical settings, allowing practitioners to anticipate survival benefits over time with greater accuracy, especially in heterogeneous patient populations where standard survival metrics might not apply as effectively [21,22,23].

However, chemotherapy, unlike targeted therapies or immunotherapies, lacks reliable pre-treatment predictors of response, which presents a challenge in limited-resource settings where only a subset of patients may derive substantial benefit from the treatment. This calls for further research to identify biomarkers or clinical indicators that can predict chemotherapy response, enabling clinicians to personalize treatment approaches even within the confines of conventional therapies. Such studies would be instrumental in developing a stratified treatment protocol for advanced NSCLC patients, optimizing survival benefits while mitigating unnecessary side effects.

The findings in this study underscore the importance of performance status, nutritional status, and chemotherapy response as crucial determinants of prognosis in advanced NSCLC. These factors can guide clinicians in identifying patients who are more likely to benefit from chemotherapy, ensuring a judicious allocation of resources in settings with limited treatment options. Nonetheless, this study has some limitations, including its single-center design and the absence of data on oncogenic driver mutations or PD-L1 expression, which could influence chemotherapy response. Moreover, follow-up computed tomography (CT) scans after two cycles of chemotherapy, as recommended in clinical guidelines, are often unfeasible in resource-constrained healthcare systems.

## 5. Conclusions

Overall, this study highlights the need to prioritize accessible prognostic markers. Good performance status, serum albumin ≥ 3.5 mg/dL, and response to first-line therapy emerged as prognostic factors for better survival in advanced NSCLC patients in resource-limited settings, where sophisticated molecular testing and advanced therapies are often unavailable. Additionally, responders demonstrated longer overall survival, as shown by RMST analysis. These findings offer a practical approach for optimizing patient outcomes through existing resources. Further multicenter research, with a focus on identifying response predictors to chemotherapy, could offer additional insights to refine treatment protocols and improve survival outcomes for patients in similar healthcare environments.

## Figures and Tables

**Table 1:** Demographic and clinical characteristics (*n* = 200).

Characteristics	*n*	%
I. Patient Factors		
Gender		
Male	99	49.5
Female	101	50.5
Age (year) *	62.4 (±9.9)	
Smoking Status		
Current/Ex-smoking	70	35.9
Never	125	64.1
NA	5	
TNM staging		
II	1	0.5
IIIa	5	2.5
IIIb	11	5.5
IV	183	91.5
PS		
ECOG 0–1	166	88.3
ECOG ≥ 2	22	11.7
NA	12	
Significant Weight Loss **		
Yes	60	30.5
No	137	69.5
NA	3	
BMI (kg/m^2^)		
<18.5	84	42.0
18.5–22.9	67	33.5
≥23	49	24.5
II. Pathological factors		
Histology		
Adenocarcinoma	130	67.0
Squamous cell carcinoma	51	26.3
Large cell carcinoma	1	0.5
NOS, others	12	6.2
NA	6	
Tumor grading		
Well differentiated	12	20.3
Moderately differentiated	13	22.0
Poorly differentiated	33	55.9
Undifferentiated	1	1.8
NA	141	
III. Pre-treatment laboratory results		
Albumin (g/dL) *	3.5 (±0.4)	
Hemoglobin (g/dL) *	11.4 (±1.9)	
WBC (cells/mm^3^) *	9562.4 (±4013.7)	
CEA (ng/mL) ***	45.4 (10.5, 195.7)	
IV. Chemotherapy Regimen		
Platinum plus Paclitaxel	109	54.5
Platinum plus Gemcitabine	91	45.5
Cycles of treatment ***	6 (4, 6)	

* Mean (±SD), ** 5% within 3 months, *** Median (IQR). Abbreviations: PS, performance status; ECOG, Eastern Cooperative Oncology Group; BMI, body mass index; NOS, not otherwise specified; NA, not available; WBC, white blood cell; CEA, carcinoembryonic antigen.

**Table 2:** Response rate by RECIST 1.1 after first-line chemotherapy (*n* = 200).

Response of Treatment	*n*	%
CR	4	2
PR	110	55
SD	60	30
PD	26	13

CR, complete response; PR, partial response; SD, stable disease; PD, progression disease.

**Table 3:** Univariate and multivariate analysis for overall survival.

		Univariate			Multivariate		
Factors	*n*	HR	95% CI	*p*-Value	HR	95% CI	*p*-Value
Age (years)							
≤60	87	1.03	0.76–1.41	0.832	1.08	0.76–1.56	0.685
>60	113	ref					
Gender							
Male	99	ref					
Female	101	0.81	0.60–1.09	0.168	1.28	0.78–2.12	0.332
ECOG							
0–1	166	0.43	0.27–0.68	<0.001	0.50	0.29–0.86	0.012
2	22	ref					
Smoking Status							
Current/Ex-smoking	70	1.34	0.98–1.85	0.070	1.02	0.62–1.68	0.926
Never	125	ref					
BMI							
<18.5	84	ref					
18.5–22.9	67	1.01	0.72–1.44	0.937	0.89	0.60–1.34	0.588
≥23	49	0.76	0.51–1.21	0.166	0.69	0.43–1.11	0.124
Histology							
Squamous	51	1.24	0.87–1.25	0.235	1.43	0.94–2.19	0.092
Non-squamous	143	ref					
Albumin							
<3.5	89	ref					
≥3.5	99	0.55	0.40–0.75	<0.001	0.60	0.41–0.89	0.010
Chemotherapy Regimen							
Platinum plus Paclitaxel	109	ref					
Platinum plus Gemcitabine	91	0.71	0.52–0.96	0.027	0.79	0.53–1.16	0.230
Responder							
Yes	114	0.60	0.44–0.82	0.001	0.57	0.39–0.83	0.003
No	86	ref					

HR, hazard ratio; CI, confidence interval.

**Table 4:** Restricted mean survival time (RMST) differences between responders and non-responders at three pre-specified landmarks (t*).

t* (Months)	Responders (*n* = 114)	Non-Responders (*n* = 86)	Effect	*p*-Value
	RMST (95% CI)	RMST (95% CI)	RMST Difference (95% CI)	
12	10.2 (9.8–10.7)	8.5 (7.8–9.2)	+1.8 (0.9–2.6)	<0.001
24	14.4 (13.1–15.7)	10.8 (9.3–12.2)	+3.6 (1.7–5.6)	<0.001
36	16 (14.1–17.8)	11.8 (9.7–13.9)	+4.2 (1.3–7.0)	0.004

CI, confidence interval; RMST, restricted mean survival time; t*, pre-specified time points for estimating the restricted mean survival time.

## Data Availability

The original contributions presented in the study are included in the article, further inquiries can be directed to the corresponding authors.
